# The first mitochondrial genome of *Megabalanus tintinnabulum* (Sessilia: Balanidae) from China: phylogeny within Cirripedia based on mitochondrial genes

**DOI:** 10.1080/23802359.2019.1688104

**Published:** 2019-11-13

**Authors:** Meiping Feng, Shiquan Lin, Chunsheng Wang, Dong Sun, Yadong Zhou, Yuanxin Bi, Kaida Xu

**Affiliations:** a Key Laboratory of Marine Ecosystem and Biogeochemistry, State Oceanic Administration & Second Institute of Oceanography, Ministry of Natural Resources, Hangzhou, PR China;; b College of Marine Ecology and Environment, Shanghai Ocean University, Shanghai, PR China;; c Marine and Fisheries Research Institute, Zhejiang Ocean University, Zhoushan, PR China;; d School of Oceanography, Shanghai Jiao Tong University, Shanghai, PR China

**Keywords:** *Megabalanus tintinnabulum*, barnacle, mitochondrial genome, Cirripedia, phylogeny

## Abstract

Here we present the complete mitochondrial genome of *Megabalanus tintinnabulum*. The genome is 15,107 bp in length with a 67.35% AT content. It contains 13 protein-coding genes (PCGs), 2 rRNAs genes, and 22 tRNAs. Both rRNAs are encoded on the light strand, as in the other crustacean and barnacle mitochondrial genomes. Besides five tRNAs are encoded on the light strand (nad1, trnV, trnL1, trnC, trnQ, and trnK). Only one PCG is encoded on the light strand (nad1), whereas the other 12 PCGs are located on the heavy strand, which is consistent with *M*. *ajax*. Phylogenetic analysis based on mitochondrial PCGs shows that *M*. *tintinnabulum* is clustered with *M*. *ajax* into a branch (BP = 100), and the group with *M*. *volcano* with high support. This study contributes to further phylogenetic analysis within Cirripedia.

*Megabalanus tintinnabulum* (Linnaeus, 1758) (Crustacea: Maxillopoda: Balanidae) is a large acorn barnacle, barrel shaped or narrowly conical, and commonly encountered in the shallow waters of both east and west coasts of India (Thiyagarajan et al. 1997). It is of tropical origin and spreads to other parts of the world attached to the hulls of ships. *Megabalanus* differs from other subgenera of the genus *Balanus* da Costa, 1778, by the possession of well-developed radii permeated by pore parallel to the basis (Beach 1972).

As of 4 September 2019, GenBank contained 31 mitochondrial genomes of Cirripedia, including 15 families, and 2 species belong to *Megabalanus*: *M*. *ajax* and *M*. *volcano*. Here we present the first complete mitochondrial genome of the *M*. *tintinnabulum*.

Specimens of *M. tintinnabulum* were collected from Yushan Island (28.88°N, 122.26°E), Ningbo City, Zhejiang Province, China. The muscle tissue isolated from the fresh specimen was immediately preserved in 95% ethanol and kept in −80 °C in Key Laboratory of Marine Ecosystem and Biogeochemistry, State Oceanic Administration, Second Institute of Oceanography, Ministry of Natural Resources (Barnacle MT-N25). DNA was extracted with QIAamp Tissue Kit (QIAGEN, Hilden, Germany) and mitochondrial DNA was amplified with a DNA REPLI-g Mitochondrial DNA Kit (QIAGEN, Hilden, Germany) as directed by the manufacturer. Library construction and sequencing were performed by Biozeron (Biozeron, Shanghai, China) using the Illumina HiSeq 4000 sequencing platform (Illumina, San Diego, CA).

The mitochondrial genome of *M. tintinnabulum* is a circular molecule which is 15,107 bp (GenBank accession number: MN 481499) in length. It contains 13 protein-coding genes (PCGs), 2 rRNAs genes, and 22 tRNAs. Total AT content of *M*. *tintinnabulum* is 67.35%. Both rRNAs are encoded on the light strand, as in the other crustacean and barnacle mitochondrial genomes (Shen et al. 2010, 2011, 2014; Tsang et al. 2015). Besides five tRNAs are encoded on the light strand (nad1, trnV, trnL1, trnC, trnQ, and trnK). Only one PCG is encoded on the light strand (nad1), whereas the other 12 PCGs are located on the heavy strand, which is consistent with *M*. *ajax* (Shen et al. 2014).

Until now three species of *Megabalanus* genus have been reported including *M. tintinnabulum*. PCGs of *M. tintinnabulum* mitochondrial genomes started with ATN. However, PCGs of the other two *Megabalanus* species have initiation codons other than ‘ATN’ (Shen et al. 2014). Furthermore, variation of initiation codon usage is observed between the three *Megabalanus* species. The *cox1* gene of *M. ajax*, *M. volcano* and *M. tintinnabulum* start with CTT, TTG, and ATT, respectively. *M. tintinnabulum* has ATA in nd3 and ATC in atp8, which is the same as *M. ajax*, whereas *M. volcano* uses ATT in both of the two corresponding genes (Shen et al. 2014).

To elucidate phylogenetic relationships of *M*. *tintinnabulum* with the other barnacles, phylogenetic tree (Figure 1) is constructed based on the PCGs with maximum likelihood using phyML version 3.0 (Guindon and Gascuel, 2003) (http://www.atgc-montpellier.fr/phyml/). A total of 29 species with 31 mitochondrial genomes from Cirripedia have been used in the phylogenetic tree, in which *Tetraclita japonica* CN and *T*. *japonica* were taken as one species (Shen et al. 2014; Shen, Chan, 2015; Shen, Tsang, 2015; Wares 2015; Baek et al. 2016; Shen, Chan, 2016; Shen, Tsoi, 2016; Shen et al. 2017; Ge et al. 2019).

**Figure 1.**
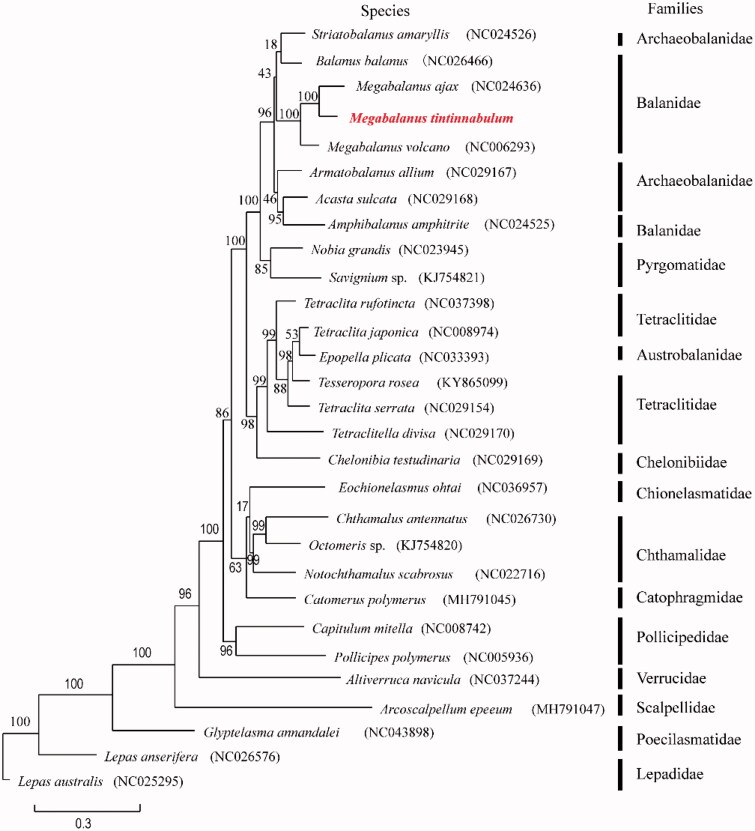
Phylogenetic tree of *Megabalanus tintinnabulum* and other mitochondrial genomes from Cirripedia based on mitochondrial PCGs.

Until 4 September 2019, the latest reference of phylogenetic tree of Cirripedia based on mitochondrial genomes used 21 mitogenomes (Ge et al. 2019), and we added 8 species for comparison in this study. The accession numbers of the added genomes were NC_029168 (*Acasta sulcata*), NC_036957 (*Eochionelasmus ohtai*), MH791045 (*Catomerus polymerus*), NC_008742 (*Capitulum mitella*), NC_037244 (*Altiverruca navicula*), MH791047 (*Arcoscalpellum epeeum*), and NC_043898 (*Glyptelasma annandalei*).

In the phylogenetic tree, *M*. *tintinnabulum* clustered with *M*. *ajax* into a branch (BP = 100), and they grouped with *M*. *volcano* with high support (BP = 100). *Amphibalanus amphitrite* as the most distantly related species within Balanidae, which was consistent with the previous results (Song et al. 2017; Cai et al. 2018). Besides, Austrobalanidae is nested within Tetraclitidae, and two families (Balanidae and Archaeobalanidae) are revealed non-monophyletic. The phylogenetic tree also supports the monophyly of three species from two families of the order Lepadiformes including *Glyptelasma annandalei* (Poecilasmatidae) and two *Lepas* species (Lepadodae), which concurs with previous studies (Pérezlosada et al. 2008; Herrera et al. 2015; Kim et al. 2019).

In this study, we present the complete mitochondrial genome sequence of *M. tintinnabulum*, which would contribute to further phylogenetic analysis of this species. Furthermore, more mitochondrial genomic data of undetermined taxa and further analysis are required to reveal phylogeny and evolution of barnacles.
